# Thermomechanical Stress Analysis of Hydrated Vital Gluten with Large Amplitude Oscillatory Shear Rheology

**DOI:** 10.3390/polym15163442

**Published:** 2023-08-17

**Authors:** Monika C. Wehrli, Anna Weise, Tim Kratky, Thomas Becker

**Affiliations:** 1 Research Group Cereal Technology and Process Engineering, Institute of Brewing and Beverage Technology, Technical University of Munich, Weihenstephaner Steig 20, 85354 Freising, Germany; 2 Department of Chemistry, Technical University of Munich, Lichtenbergstraße 4, 85748 Garching, Germany

**Keywords:** LAOS rheology, Lissajous–Bowditch, nonlinear viscoelasticity, biopolymer, denaturation, CLSM

## Abstract

Vital gluten is increasingly researched as a non-food product for biodegradable materials. During processing, the protein network is confronted with increased thermal and mechanical stress, altering the network characteristics. With the prospect of using the protein for materials beyond food, it is important to understand the mechanical properties at various processing temperatures. To achieve this, the study investigates hydrated vital gluten under thermomechanical stress based on large amplitude oscillatory shear (LAOS) rheology. LAOS rheology was conducted at increasing shear strains (0.01–100%), various frequencies (5–20 rad/s) and temperatures of 25, 45, 55, 65, 70 and 85 °C. With elevating temperatures up to 55 °C, the linear viscoelastic moduli decrease, indicating material softening. Then, protein polymerization and the formation of new cross-links due to thermal denaturation cause more network connectivity, resulting in significantly higher elastic moduli. Beyond the linear viscoelastic regime, the strain-stiffening ratio rises disproportionately. This effect becomes even more evident at higher temperatures. Lacking a viscous contribution, the highly elastic but also stiff network shows less mechanical resilience. Additionally, at these elevated temperatures, structural changes during the protein’s denaturation and network shrinkage due to water evaporation could be visualized with confocal laser scanning microscopy (CLSM).

## 1. Introduction

In the food industry, vital gluten is an established additive for baking, plant-based meat alternatives and a binding agent for a wide variety of foods [1,2,3,4,5]. Meanwhile, its potential as a relatively abundant and low-cost non-food material for biodegradable plastics is increasingly researched due to the protein´s hydrophobic properties and its unique viscoelastic nature [1,6,7,8,9,10,11,12,13]. The fabrication of such a biopolymer with desired textural properties often involves processes such as extrusion, mixing and (thermo-) molding. These processes put the material under both thermal and mechanical stress at the same time [6,7,12,14,15,16,17,18]. Although not all mechanisms of increased mechanical stress on the gluten protein network are understood in detail, it is known that overmixing leads to a weaker and less resistant protein network on a macroscopic scale [19]. On a molecular scale the extended mechanical input alters the secondary structure of the protein. Namely, the amount of β-turns can decrease while β-sheets are formed [20,21,22]. Furthermore, the breakdown of disulfide bonds, intramolecular and intermolecular bonds and the disruption of hydrogen bonding can weaken the protein network and lead to a smaller size of protein aggregates as well as higher protein extractability [20,23,24]. Changes in the molecular structure of the protein can also be caused by the application of heat, up to the point of denaturation [25,26,27,28,29]. The thermostability of dry gluten powder is very high with a glass transition temperature ($T_{g}$) ranging between 155 °C and 164 °C as determined by dynamic scanning calorimetry (DSC) [30,31]. In contrast, hydrated gluten has a $T_{g}$ in the range of 38 to 60 °C [32,33], which depends on the gliadin to glutenin ratio. The smaller, globular gliadin fraction demonstrates greater thermal stability in comparison to the larger, filamentous glutenin fraction [29,34,35]. The hydrated gluten network shows two distinct temperature ranges occurring at 56–64 °C and 79–81 °C, at which thermal effects become visible in the rheological network properties [30]. The literature suggests a loss of α-helical structures, formation of new β-structures and intermolecular β-sheet aggregation above temperatures of 65 °C [30,36,37]. Above 70 °C, glutenin polymerization is observed and an increasing number of cross-links leads to a highly branched network [27,38]. Rahaman et al. (2016) found that heating to 100 °C ultimately causes a shift from stretched and flexible form to a helical and random coiled structure, which alters the tertiary and quaternary structure of the protein [37]. The structural rearrangements due to thermal stress lead to a decreased protein extractability, especially for α- and γ-gliadin. They also lead to a decrease of free thiol content due to disulfide bonding-driven cross-linking of glutenin molecules, mainly via thiol oxidation, and to a lesser extent, due to thiol-disulfide interchange reaction [37,39]. The changes of this highly polymerized protein network can significantly impact the material behavior, affecting its elastic and viscous characteristics as well as its mechanical resilience.

To shed light on the effects of changes in the protein´s structure on the network characteristics and viscoelastic behavior of the biopolymer, numerous studies employ rheological methods [29,40,41,42,43,44,45]. Some studies, e.g., the work by Angioloni and Dalla Rosa (2005), have also explored the thermomechanical properties of wheat dough within the linear viscoelastic regime (LVE) [46]. While many studies primarily focus on assessing the gluten network´s behavior in the LVE, it is imperative to explore the material at higher shear strains, considering the substantial mechanical input on the network during processing [19,47]. Large amplitude oscillatory shear (LAOS) rheology presents an effective approach to study materials at high shear strains. Here, the strain amplitude $\gamma_{0}$ is increased while maintaining a constant angular frequency ω. This increases the shear strain imposed on the sample. The LVE is consequently left toward the nonlinear region where the amplitude of the response is not directly proportional to the amplitude of the applied ingoing shear strain [48]. With the nonlinear response not being fully sinusoidal, higher harmonics can contribute to the interpretation of the rheological data [49]. To quantify the oscillatory stress σ(t) to the sinusoidal strain input $\gamma \left(t\right)=\gamma_{0}\sin\left(\omega t\right)$, the Fourier transform of σ(t) is commonly used. The total oscillatory stress σ(t) is described as a Fourier series, taking into account both elastic and viscous scaling contributions reflected by sine and cosine terms, respectively [49,50,51,52,53]:

(1) $$\sigma \left(t,\omega ,\gamma_{0}\right)=\gamma_{0}\sum_{nodd}\{G_{n}^{\prime }\left(\omega ,\gamma_{0}\right)\sin\left(n\omega t\right)+G_{n}^{\prime \prime }\left(\omega ,\gamma_{0}\right)\cos\left(n\omega t\right)\}$$

${G\prime }_{n}$ and ${G\prime \prime }_{n}$ are the elastic and viscous moduli. Due to the symmetry of the experiment and the nature of the analyzed material only fundamentals (n=1) and odd higher harmonics $\left(n=3,\;5,\;\ldots \right)$ appear [54,55,56,57,58]. From this, various parameters describing the non-linear behavior of the material can be derived, such as the strain stiffening ratio S and the shear thickening ratio T, which describe the nonlinearity per oscillation cycle (intra-cycle). The nonlinearities can also be visualized using Lissajous–Bowditch curves. Plotting the shear strain versus the stress response represents the elastic Lissajous–Bowditch curves, where purely elastic materials are characterized by a diagonal line while purely viscous materials result in a circular trajectory [50].

Previous studies have either investigated the impact of thermal stress [17,42,59,60,61,62,63,64] or the mechanical stress using LAOS rheology [53,65,66,67,68,69,70] on gluten. However, there remains a knowledge gap regarding the combined effect of thermomechanical stress on the gluten network. This study investigates the combined thermal and mechanical resilience of the protein with LAOS rheology at various temperatures below, during and above thermal denaturation of the protein. A more profound understanding of the network changes during thermal stress is revealed through confocal laser scanning microscopy (CLSM). By combining thermal stress with LAOS rheology, this research provides valuable insights into the behavior of gluten beyond the linear viscoelastic regime (LVE), offering a more comprehensive understanding of its response closer to realistic processing conditions.

## 2. Materials and Methods

### 2.1. Vital Gluten Preparation and Rehydration

Vital gluten was produced from wheat flour (type 550, Rosenmühle GmbH, Ergolding, Germany) using a food processor with a kneading hook (MUM4405, Robert Bosch GmbH, Stuttgart, Germany), which was equipped with a perforated bowl and a polyester cloth with 80 µm mesh size. Water-soluble components of the flour were washed out with a continuous supply of desalted water. The remaining wet gluten was freeze-dried for 72 h (Beta 1-8 LSCplus, Martin Christ Gefriertrocknungsanlagen GmbH, Osterode am Harz, Germany) and then milled in an ultra-centrifugal mill (Retsch GmbH, Haan, Germany) at a constant speed of 12,000 rpm and a sieve with a mesh size of 250 µm to obtain vital gluten powder. The residual starch content of the vital gluten samples was 3.11 ± 0.16% (4 measurements, total starch enzyme kit, Megazyme International Ireland, Ltd., Wicklow, Ireland). For further measurements, the vital gluten was rehydrated following the solvent retention capacity (SRC) method (AACC 56-11.02 [71]). The SRC of the gluten was 133.8 ± 3.6% (59 measurements, 14% moisture basis).

### 2.2. Rheological Measurements

An MCR 502 rheometer from Anton Paar equipped with a CTD 180 heating chamber and parallel riffled plate geometry (upper plate: PP25/P22 2; lower plate: L-PP25/CTD 600/P; diameter: 25 mm) was used (RheoCompass software version 1.31, Anton Paar GmbH, Graz, Austria). The rehydrated gluten was placed between the plates, and the edges were trimmed and covered with a layer of paraffin oil to prevent drying during measurement. Then, the sample was relaxed for 20 min while reaching the measuring temperature before a rheological measurement was conducted. As $G\prime_{1}>G{\prime \prime }_{2}$ in the measurements, the material can be described as a gel, making the plate–plate setup suitable for gluten samples [72,73,74]. All measurements were performed in triplicate.

#### 2.2.1. Large Amplitude Oscillatory Shear (LAOS) Rheology

LAOS measurements at a constant temperature of 25, 45, 55, 65, 70 or 85 °C were each conducted at frequencies of 5, 8, 10, 15 and 20 rad/s at shear strains γ between 0.01 and 100%. The storage $G\prime_{n}$ and loss moduli $G{\prime \prime }_{n}$ of the fundamental (n=1) and third harmonic (n=3) were used for data evaluation and calculation of the strain stiffening ratio S and shear thickening ratio T. Undesirable effects such as wall slip were excluded as even harmonics were not observed in any measurement [55,57,58].

#### 2.2.2. Determination of the Linear Viscoelastic Regime (LVE)

To determine the LVE of gluten at various temperatures, the fundamental elastic response $G_{1}^{\prime }$ of the LAOS experiment was evaluated. The elastic limit yield stress was defined as the point at which $G_{1}^{\prime }$ deviated more than 5% from the plateau value of the LVE region [48].

### 2.3. Confocal Laser Scanning Microscopy (CLSM)

The protein network of the hydrated gluten was stained with a layer of Rhodamine B (0.01% *w*/*v* aqueous solution). The dye does not affect the gluten microstructure [75]. A confocal laser scanning microscope (Nikon D-Eclipse C1, Nikon, Amsterdam, The Netherlands) equipped with a 20× objective, and 543 nm laser was used for imaging of the gluten protein network. The temperature during the measurement was increased with a heating stage (mK1000–INS1201033, INSTEC, Boulder, CO, USA) from 20 °C to 85 °C at a rate of 10 °C/min.

### 2.4. Analysis and Statistical Evaluation

For each measured parameter, the arithmetic mean and standard deviation were calculated from multiple measurements. The error originating from the sample was assumed to be dominant, while the error from the rheological measurement was neglected. Further evaluation was completed with Origin 2021b (OriginLab Corporation, Northampton, MA, USA) and IGOR Pro 6.37 (WaveMetrics, Portland, OR, USA).

## 3. Results and Discussion

### 3.1. Linear Rheological Behavior of Vital Gluten at Various Temperatures

**Figure 1:**
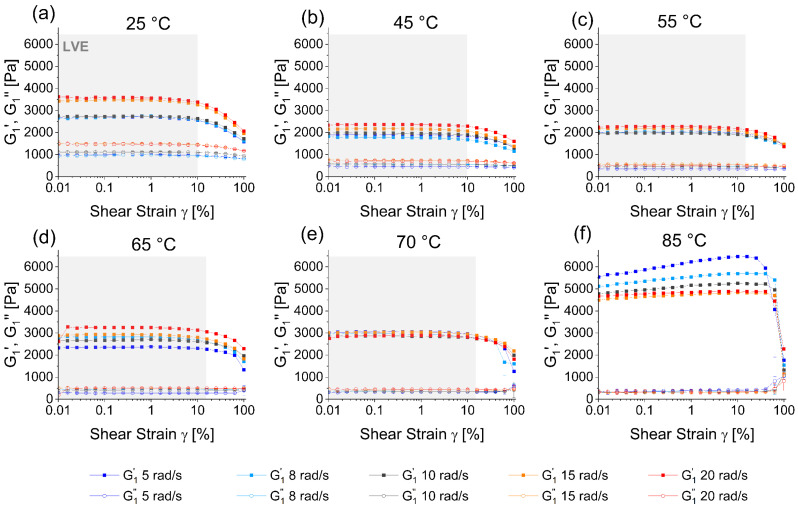
Fundamental response of the LAOS measurement with storage modulus ($G_{1}^{\prime }$) shown as full squares and loss modulus ($G_{1}^{\prime \prime }$) as hollow circles at all measuring temperatures (**a**–**f**) and frequencies (color-coded). The linear viscoelastic regime (LVE) is marked as gray areas.

During production and subsequent processing, gluten often undergoes both thermal and mechanical stress. To see the effect of thermomechanical stress on the protein, LAOS rheology measurements at a shear strain increasing from 0.01 to 100% were conducted at various temperatures. Figure 1 presents the fundamental elastic ($G_{1}^{\prime }$) and viscous ($G_{1}^{\prime \prime }$) response of hydrated vital gluten at temperatures of 25 °C to 85 °C (a–f) and angular frequencies of 5 (dark blue), 8 (light blue), 10 (black), 15 (orange) and 20 (red) rad/s (Supplementary Figure S1 presents the data per angular frequency with color-coded temperature). Throughout all temperatures and angular frequencies, the elastic moduli are significantly higher than the viscous moduli, which makes gluten a highly elastic gel [72,73,74]. The viscoelastic moduli increase slightly with higher angular frequency at a given temperature with few deviations from this trend: At 70 °C the effect is very weak and at 85 °C the opposite trend can be observed. According to the literature, gluten has two temperature ranges (56–64 °C and 79–81 °C) where a change associated with thermal denaturation can be seen both in the protein structure as well as in the network properties [30]. At 85 °C where the protein is above its denaturation temperature, the increased number of cross links and higher polymerization make the network more compact, which may make the measurability of the highly elastic material at too low angular frequencies more difficult, resulting in higher measured elastic moduli.

The elastic ($G_{1}^{\prime }$) and viscous ($G_{1}^{\prime \prime }$) moduli leave the linear viscoelastic regime (LVE), marked as gray areas in Figure 1, at high shear strains. For measuring temperatures of 25–45 °C the LVE is left at ca. 10% and for higher temperatures of 55–70 °C the LVE expands to a shear strain of ca. 15%. As the curves at 85 °C increase constantly without reaching a plateau, the same calculation of the LVE is not possible. Although the LVE extends to higher shear strains with increased temperatures, the gluten network demonstrates reduced resilience to mechanical stress. At elevated temperatures ≥ 70 °C (Figure 1e,f) the curves of the viscoelastic moduli become very close and unsteady at high shear strains. At 85 °C network collapse becomes evident when $G_{1}^{\prime }$ and $G_{1}^{\prime \prime }$ cross at a shear strain of 100%. Rahaman et al. (2016) found that a combination of high shear rate and temperature resulted in an unfolding of the protein, which exposed hydrophobic residues and disrupted secondary, tertiary and quaternary structures [37]. This structural disruption of the protein likely contributes to the diminished resilience of the gluten network at elevated temperatures.

In the LVE at 25 °C, $G_{1}^{\prime }$ and $G_{1}^{\prime \prime }$ range from 2700 to 3500 Pa and from 1000 to 1500 Pa, respectively, depending on the angular frequency. Typical plateau values reported in the literature are in the range of 1000–9000 Pa for $G_{1}^{\prime }$ and 200–4000 Pa for $G_{1}^{\prime \prime }$ [47,53,65,66]. The large variations can originate from differing sample characteristics due to environmental impact, selection of raw material and processing conditions during production [2,23,76,77,78,79,80,81,82]. When heating the protein to 45 °C, both $G_{1}^{\prime }$ and $G_{1}^{\prime \prime }$ values decrease. Further heating to 55 °C does not show any distinct effect. The elastic modulus increases again at higher thermal stress, while the viscous modulus remains at low values regardless of the measuring temperature. The material measured at 85 °C shows the clearest effect to thermal stress with elastic moduli ranging from 4500 to 5500 Pa. The initial decrease of the viscoelastic moduli upon heating to 45 °C compared to the curves at 25 °C may be explained by material softening [83]. The low $G_{1}^{\prime }$ at 55 °C and increase at higher temperatures is associated with protein aggregation [29], and on a structural level a loss of α-helices and the formation of intermolecular β-sheets is reported when heating the protein above 40 °C [36]. At 85 °C the protein has experienced complete thermal denaturation. The high elastic moduli of the protein are presumably caused by polymerization, in the course of which new intermolecular disulfide cross links are formed, mainly via thiol oxidation [26,29,39,60,84].

Figure 1 further shows that at 25 °C the viscous moduli decrease slightly at high shear strains (Figure 1a). However, at temperatures of 65 °C and above (Figure 1d–f), the viscous moduli show an increasing trend when shear strains exceed 40%. The gliadin-fraction, which is more thermally stable than the glutenin fraction, is mainly responsible for the viscous properties of the gluten network [85]. At this point the material is past the first of two temperature ranges (56–64 °C and 79–81 °C), where denaturation effects show a change in network properties [30]. The increased viscous material behavior at high shear strains could, therefore, be attributed to the gliadin fraction being predominantly intact at higher temperatures, while at these temperatures mainly the glutenin fraction is affected [39]. Regarding the use of the biopolymer in food and non-food products, the partial denaturation can be used to an advantage when only the elastic properties are required and the material needs to maintain its form under physical stress.

### 3.2. Non-Linear Rheological Behavior of the Gluten Network at Various Temperatures

**Figure 2:**
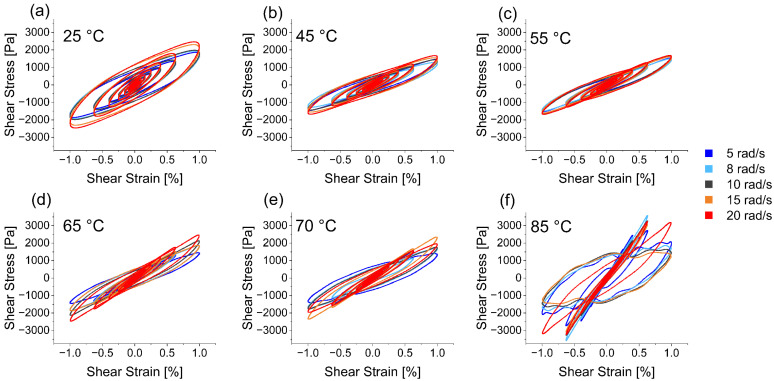
Elastic Lissajous–Bowditch curves at temperatures of (**a**) 25 °C, (**b**) 45 °C, (**c**) 55 °C, (**d**) 65 °C, (**e**) 70 °C, and (**f**) 85 °C and at angular frequencies of 5 to 20 rad/s (color-coded).

In addition to addressing the linear rheological response of a material mainly based on the fundamentals, LAOS rheology is also a suitable method to address non-linear properties of a material beyond the LVE. Figure 2 shows the elastic Lissajous–Bowditch curves acquired at temperatures from 25–85 °C (a–f) and angular frequencies ranging from 5 to 20 rad/s (color-coded). Each curve represents the plot of the stress response versus strain for a given maximum strain reflecting the intra-cycle behavior of the material. Mainly the effects of the last 3–4 measuring cycles at high shear strains are visible and can therefore be described, where the material response has already left the LVE. The elliptical shape of the curves proves a viscoelastic behavior regardless of the temperature or angular frequency. The narrow appearance of the curve is characteristic for a dominant elastic contribution which goes in line with higher elastic moduli compared to the viscous moduli. While the ratio from elastic to viscous properties affects the shape of the curve, the extent of the elastic contribution is influenced by the frequency of the applied strain. A counterclockwise rotation of the Lissajous–Bowditch curves with increasing angular frequency is particularly visible in the curves at 25 °C (Figure 2a), 65 °C (Figure 2d) and 70 °C (Figure 2e with one outlier). This nonlinear response of the material seen in Figure 2 evocates a strain-stiffening behavior at high shear strains [65,70]. Note that this does not interfere with the previously described material softening in Figure 1, where the whole range of imposed shear strain was addressed.

Changes in the material characteristics during heating can be categorized into four groups: At 25 °C (Figure 2a) the material shows the highest viscous contribution at high shear strains, evident from the widest ellipses. The curves recorded at 45 °C and 55 °C (Figure 2b,c) display very similar shapes, with a reduced viscous contribution indicated by narrower curves. The curves measured at 65 °C and 70 °C (Figure 2d,e) exhibit a pointier shape. Lastly, the material measured at 85 °C (Figure 2f) shows the most pronounced reaction at high shear strains. As mentioned before, the material at 85 °C is less resilient to mechanical stress and a network collapse at 100% shear strain becomes evident with the curves following an irregular pattern. The four categories correspond to the previously discussed temperature ranges at which gluten shows denaturation effects. The curves at 45 °C and 55 °C are still below the temperature ranges but compared to 25 °C they exhibit material softening. The protein is in between the two temperature ranges at 65 °C and 70 °C and above thermal denaturation temperature at 85 °C.

**Figure 3:**
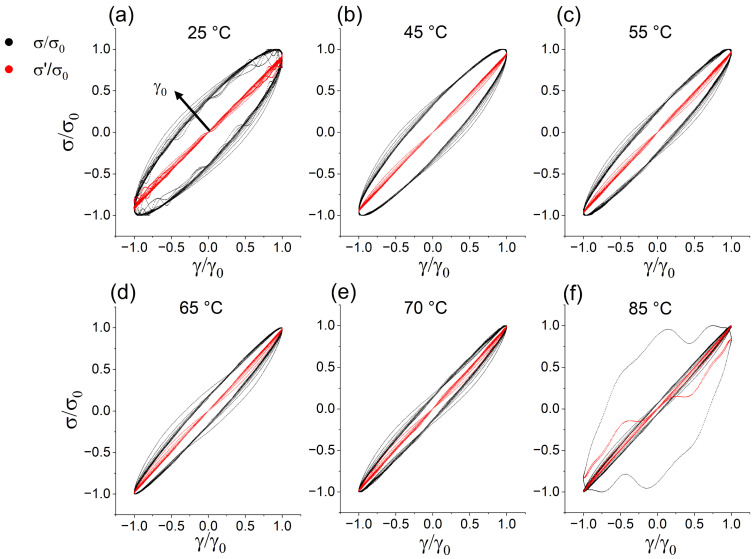
Normalized elastic Lissajous–Bowditch curves at temperatures from 25 to 85 °C (**a**–**f**) measured at an angular frequency of 10 rad/s. The arrow indicates the curves acquired at higher maximum shear strain $\gamma_{0}$ while the curves represent the total stress (black) and elastic stress σ′ (red).

To address not only the material behavior at the highest shear strains but also the LVE and the onset of nonlinear behavior, the elastic Lissajous–Bowditch curves are normalized in both dimensions with respect to their maximum ($\gamma /\gamma_{0}$ and $\sigma /\sigma_{0}$). For clarity, only the curves at 10 rad/s for total stress (black) and elastic stress σ′ (red) are shown in Figure 3 as the trends are comparable at all angular frequencies. (For overall normalized elastic and viscous Lissajous–Bowditch curves at all frequencies and temperatures, see Supplementary Figures S2 and S3). In the LVE, the curves are fully symmetric ellipses (mirror symmetry with respect to two perpendicular axes). With increasing temperature, they become narrower, proving an elastically dominated behavior with less viscous contribution to the material. This corresponds to the findings in Figure 1, where gradually lower values for the viscous moduli were measured at higher temperatures. When the curves lose the mirror symmetry at higher shear strains above 10–15%, the material leaves the LVE and non-linear effects are observed. The previously described four categories can be identified, too. The normalized elastic Lissajous–Bowditch curves display the highest viscous contribution. At 45 °C and 55 °C the curves look very similar to one another with notably less viscous properties. The curves at 65 °C and 70 °C are also very similar to each other and have a pointier shape than the curves at lower temperatures. At 85 °C the curves remain in a very narrow ellipse, except for the last measuring cycle at 100% shear strain, where the uneven trajectory implicates a material collapse. At this stage, the protein experienced thermal denaturation resulting in a highly cross-linked and polymerized network. The lack of viscous contribution makes it less flexible and the structural integrity of the protein suffers from a disruption of disulfide and hydrogen bonds while the ratio of β-sheets to β-turns increases [20,21,22,23,24].

### 3.3. Quantification of the Nonlinear Rheological Properties

Common parameters to quantify nonlinearities in the stress response of a material are the strain stiffening ratio (S) and the shear thickening ratio (T). Only minor shear thinning effects (T<0) can be observed for high shear strains. The effect is not very pronounced for gluten networks, which is in good agreement with the literature describing this effect at room temperature [53,82] (see Supplementary Figure S4 for plots with T).

**Figure 4:**
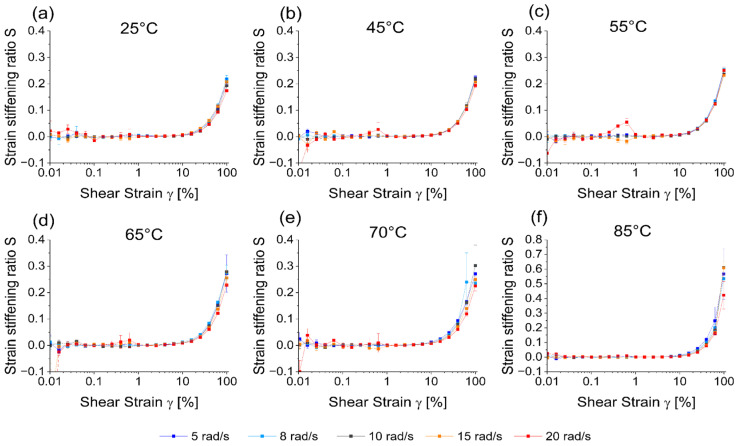
Strain stiffening ratio S at temperatures from 25 to 85 °C (**a**–**f**) and angular frequencies of 5–20 rad/s (color-coded). The scale the ordinate in Figure 4f is different compared to the others for better readability of all graphs.

Figure 4 presents S at different temperatures (a–f) and angular frequencies (color-coded). For better readability, the scale at Figure 4a–e is different from the scale of Figure 4f. Deviating values at ~1% shear strain measured at 20 rad/s are a measuring artifact induced by the experimental setup. The material does not display any nonlinearities at lower shear strains when S=0 or T=0. This is another way the LVE can be determined, which also spans up to ca. 10% for 25–45 °C and ca. 15% for all higher temperatures. While the calculation of the LVE was previously not possible at 85 °C, this is no issue here. In addition, no influence from the angular frequency of the measurement on S is observed in Figure 4. S>0 describes an intra-cycle strain-stiffening of the material under mechanical stress. The strain-stiffening effect becomes disproportionately stronger with higher shear strain. The values of S at room temperature and its positive correlation with higher shear strains are consistent with previous research [53,82]. At elevated temperatures, the disproportional increase of S at high shear strains becomes even more pronounced. This would indicate that the gluten network has a stronger nonlinear character at high temperatures. Figure 4 also complements the findings from Figure 2 where the counterclockwise rotation of the Lissajous–Bowditch curves would indicate strain-stiffening. While in Figure 2 this behavior was not easily visible for all plots, the effect is very clear in Figure 4. The high strain-stiffening might also explain the lower mechanical resilience and network collapse of the polymer at 85 °C. With higher temperature, the protein denaturation is more pronounced and thus a higher degree of polymerization, higher number of new intermolecular disulfide cross-links, effect a stiffer and more elastic network [20,21,22,23,24,26,29,39,60,84]. In the absence of the viscous component, the stiff and interconnected protein network seems stronger. However, the critical shear strain at which the network breaks down is reached earlier due to its reduced flexibility.

### 3.4. Optical Evaluation of Gluten during Thermal Stress

**Figure 5:**
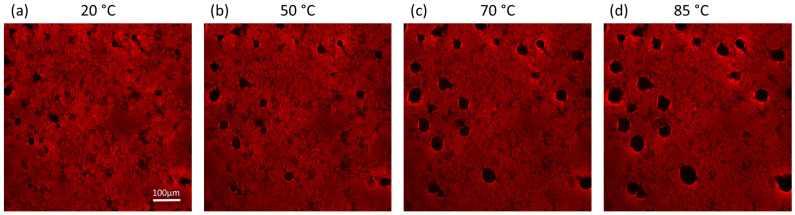
CLSM images of the gluten gel network at 20, 50, 70 and 85 °C (**a**–**d**). The protein in red is dyed with Rhodamin B.

CLSM images in Figure 5 visualize the effect of temperature between 20 °C and 85 °C on the gluten network. The gluten protein network appears in red due to dyeing with Rhodamin B while heating at 20 °C (a), 50 °C (b), 70 °C (c) and 85 °C (d) (see Supplementary Figure S5 for all temperatures). From 20 to 50 °C (Figure 5a,b), the rough surface of the protein matrix appears relatively homogeneous and branched with some small dark spots where the network is not connected. Above 70 °C and beyond (Figure 5c,d), the sample surface appears smoother in the images. At these temperatures, the previously described rheological measurements showed that the network gets more elastic, stiffer and less resilient. This can indicate a polymerization of the network with high temperatures, which could be represented in the CLSM images by the smoother surface that appears to be more compact. The transformation could be attributed to a combined effect of heat-induced polymerization, protein denaturation and glutenin aggregation [65,66]. Moreover, the expansion of the few holes in the network along with its shrinkage can be caused by water evaporation from the gluten gel at elevated temperatures. The evaporation process is driven by the exponential increase in water vapor pressure with temperature following the Clausius–Clapeyron equation. This leads to a decrease of moisture content from 33% before and 20% after heating to 85 °C for a total time of 65 min. The increasing water vapor pressure also causes expanding holes in the network. Note that the growing holes in the network are not newly formed but originate from pre-existing small holes which then all become a similar size. While the increased compactness of the network is probably interconnected with the higher elastic modulus, the larger holes and lower moisture content might account for the diminished mechanical resilience at high shear strain. It is evident how important network connectivity and the distribution and presence of water-pockets are for network characteristics. The interplay between network structure, protein denaturation and water distribution at different temperatures determines the material´s rheological properties and paves the way toward tailor-made biopolymers.

## 4. Conclusions

In this study, the effect of thermal and mechanical stress on the hydrated vital gluten network was assessed using LAOS rheology and CLSM imaging. The rheological results show four categories of material responses to the thermomechanical stress, which are connected to two denaturation ranges of gluten previously described in the literature. While the elastic component depends on these categories, the viscous component is significantly diminished in the linear viscoelastic regime (LVE) with increasing temperature. One category represents the protein at room temperature and the second one describes material softening without thermal denaturation where both viscous and elastic moduli decrease. A third category shows samples between the two denaturation ranges where the elastic moduli again increase, and one category is for the fully denatured and polymerized protein network with very high elastic and very low viscous moduli in the LVE. The analysis of the nonlinear rheological response reveals that the material experiences only minor shear thinning but disproportionally strong intra-cycle strain-stiffening at high shear strains starting from 10 to 15%. High thermal stress ultimately results in a polymerized stiff protein network with a high amount of cross links that show low mechanical resilience—as without the viscous component, it lacks flexibility. CLSM imaging reveals a more compact network at high temperatures due to polymerization of the material and network shrinkage driven by water evaporation. With this knowledge, it is possible to deliberately design the gluten polymer by applying specific thermal pre-treatments to achieve desired elastic behavior and mechanical flexibility.

## Data Availability

Data can be shared upon contacting the authors. This is part of our current internal publishing policies.

## Supplementary Materials

The following supporting information can be downloaded at: https://www.mdpi.com/article/10.3390/polym15163442/s1.
