# Supplementary material for: Thermomechanical Stress Analysis of Hydrated Vital Gluten with Large Amplitude Oscillatory Shear Rheology
Source: Polymers (Basel). 2023 Aug 17;15(16):3442. doi: 10.3390/polym15163442 (PMC10459017; doi:10.3390/polym15163442)
Supplement: Supplementary file 1 [file polymers-15-03442-s001.zip › polymers-2509011-supplementary.pdf]

Supplementary material

Thermomechanical Stress Analysis of Hydrated Vital Gluten with Large Amplitude Oscillatory Shear Rheology

Monika C. Wehrli [1,*], Anna Weise [1], Tim Kratky [2] and Thomas Becker [1]

[1] Research Group Cereal Technology and Process Engineering, Institute of Brewing and Beverage Technology, Technical University of Munich, Weihenstephaner Steig 20, 85354 Freising, Germany

[2] Department of Chemistry, Technical University of Munich, Lichtenbergstraße 4, 85748 Garching, Germany

* Correspondence: monika.wehrli@tum.de

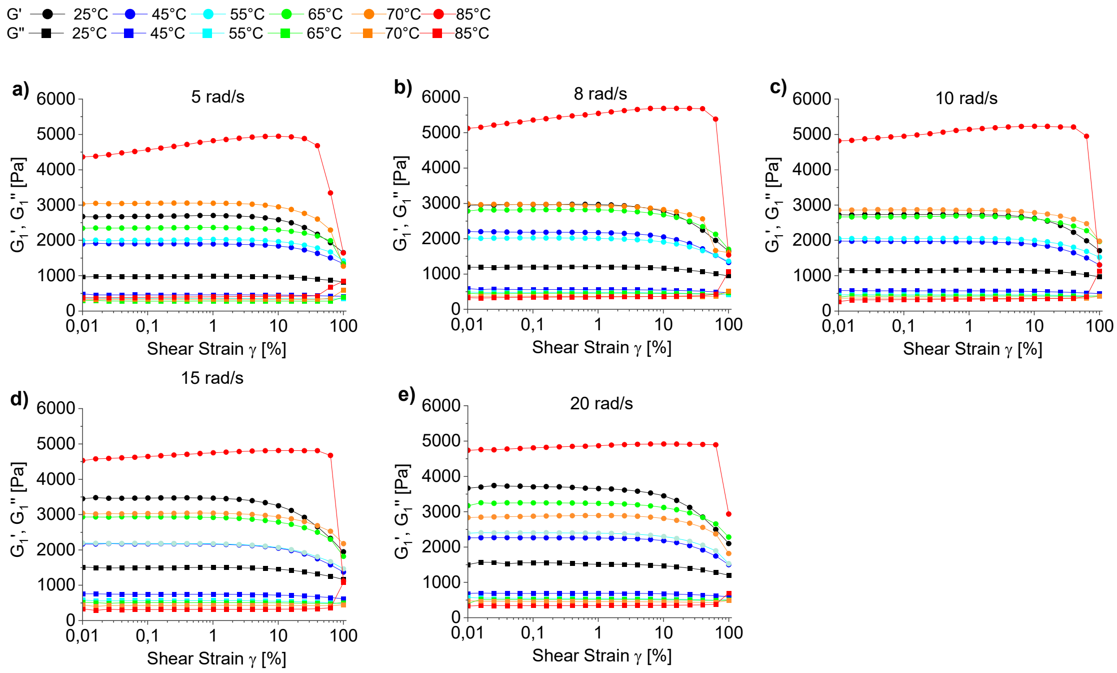

Figure S1: Fundamental rheological response of vital gluten measured at angular frequencies of 5 – 20 rad/s (a – e) and at temperatures of 25 – 85 °C (color coded).

Table S1. Critical shear strain for each conducted temperature.

T [°C]	25	45	55	65	70	85
$\sigma_{\text{LVE left}}$ [%]	10.1– 15,9	10.1 – 15,9	15.9 – 25.2	15.9 – 25.2	15.9 – 25.2	No clear LVE plateau for calculation

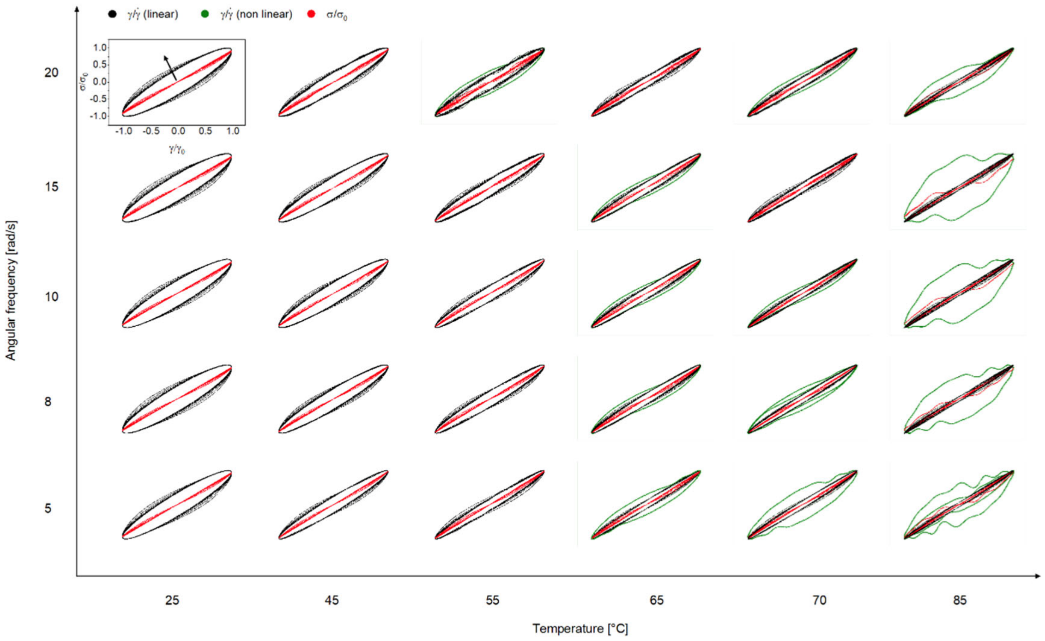

Figure S2: Normalized elastic Lissajous-Bowditch curves of hydrated gluten for all measured temperatures and angular frequencies.

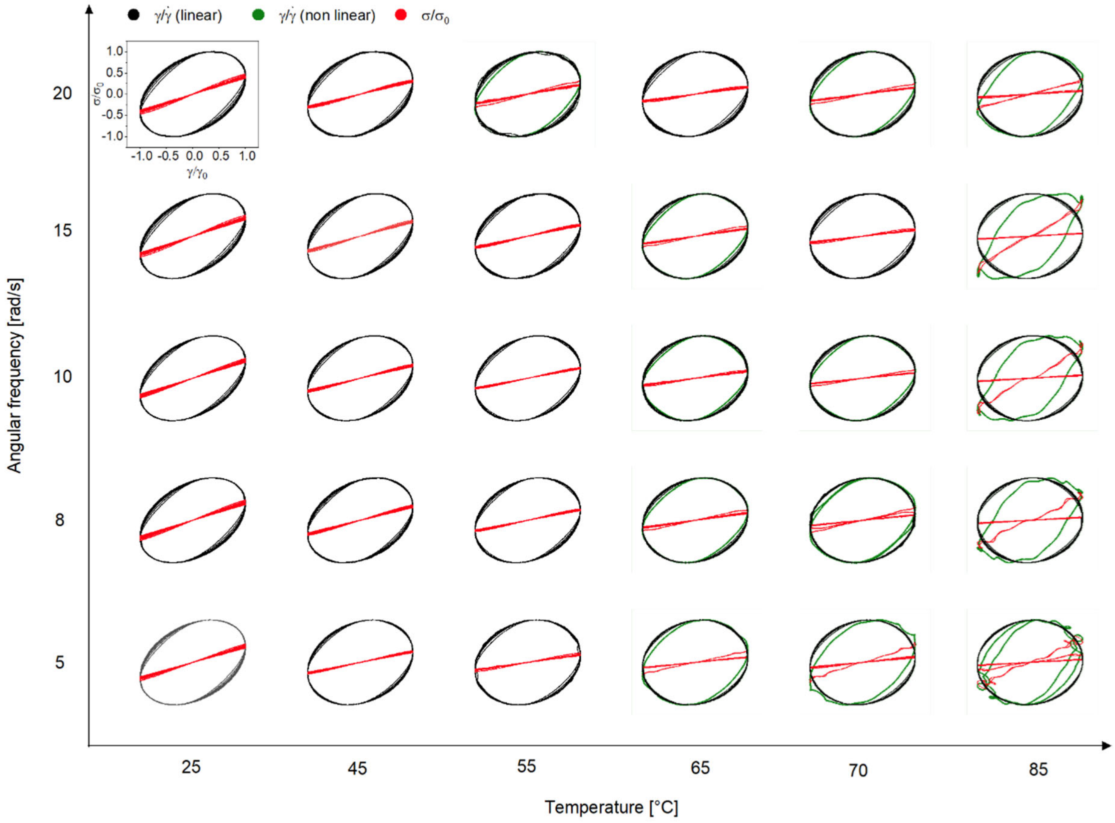

Figure S3: Normalized viscous Lissajous-Bowditch curves of hydrated gluten for all measured temperatures and angular frequencies.

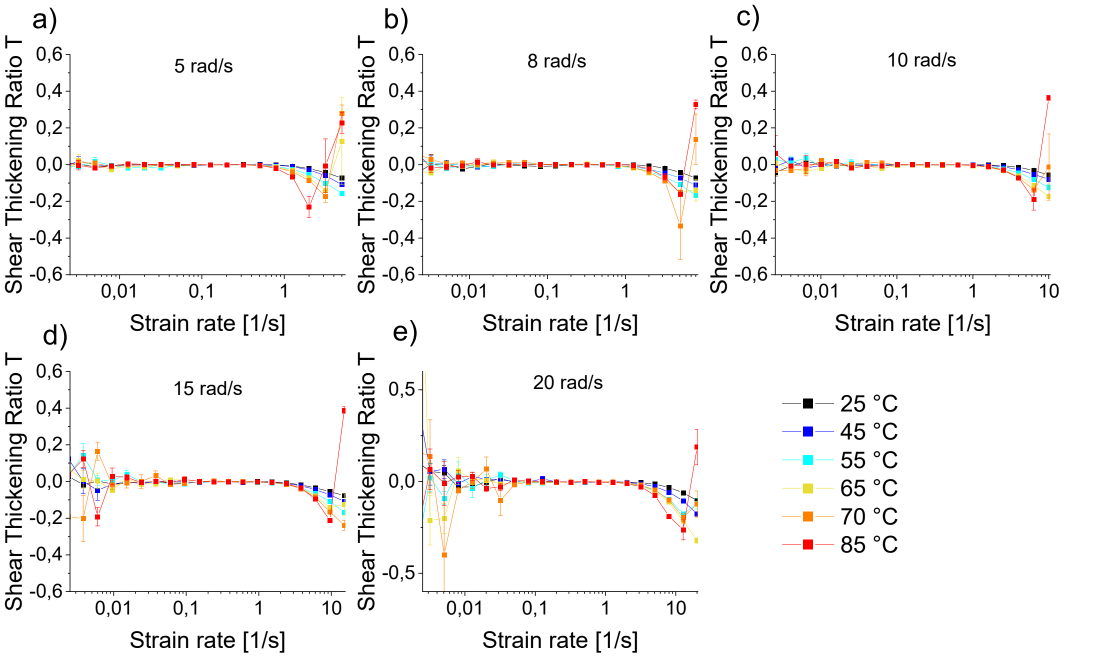

Figure S4: Shear thickening ratio T of hydrated gluten measured at various temperatures (color coded) and angular frequencies of 5 – 20 rad/s (a – e).

T is temperature- and frequency- dependent: With higher measuring temperature, the values of T are lower and the material becomes shear thinning above a strain rate of 1/s (for 5 rad/s) to 3/s (for 20 rad/s).

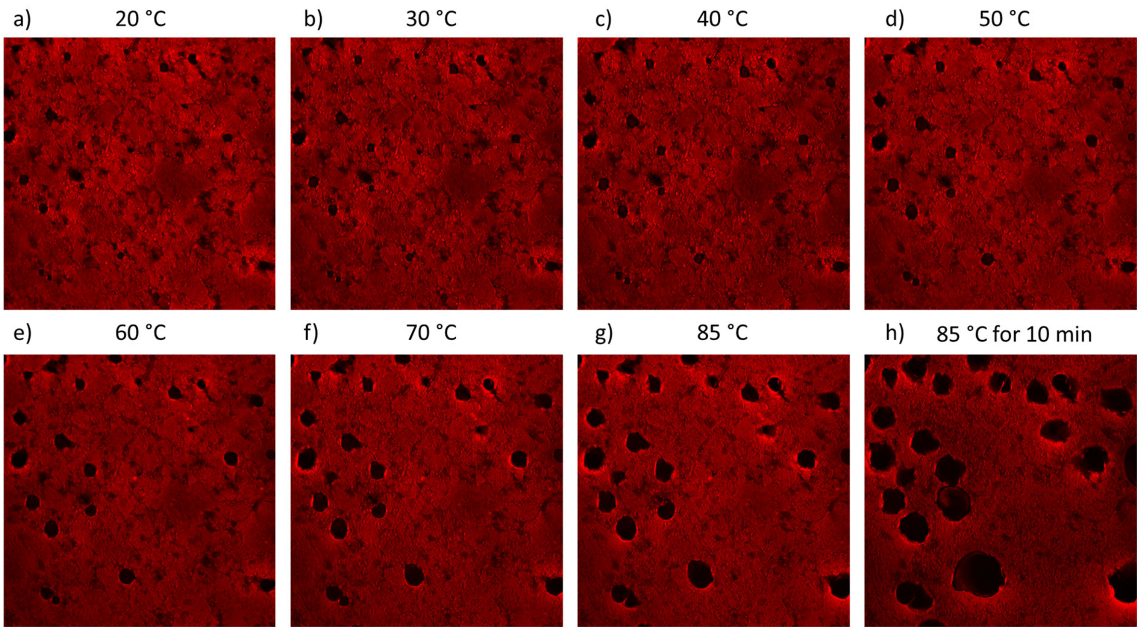

Figure S5: CLSM images of hydrated gluten at measuring temperatures of 20 – 85 °C (a – h).
